# Electrodiagnostic tests of the visual pathway and applications in neuro-ophthalmology

**DOI:** 10.1038/s41433-024-03154-6

**Published:** 2024-06-11

**Authors:** Antonio Calcagni, Magella M. Neveu, Neringa Jurkute, Anthony G. Robson

**Affiliations:** 1 Department of Electrophysiology, Moorfields Eye Hospital, London, UK; 2 Institute of Ophthalmology, University College London, London, UK; 3 National Institute of Health Research Biomedical Research Centre at Moorfields Eye Hospital and the UCL Institute of Ophthalmology, London, UK; 4 Department of Neuro-ophthalmology, Moorfields Eye Hospital, London, UK; 5 Department of Neuro-ophthalmology, The National Hospital for Neurology and Neurosurgery, University College London Hospitals NHS Foundation Trust, London, UK

**Keywords:** Signs and symptoms, Diseases

## Abstract

This article describes the main visual electrodiagnostic tests relevant to neuro-ophthalmology practice, including the visual evoked potential (VEP), and the full-field, pattern and multifocal electroretinograms (ffERG; PERG; mfERG). The principles of electrophysiological interpretation are illustrated with reference to acquired and inherited optic neuropathies, and retinal disorders that may masquerade as optic neuropathy, including ffERG and PERG findings in cone and macular dystrophies, paraneoplastic and vascular retinopathies. Complementary VEP and PERG recordings are illustrated in demyelinating, ischaemic, nutritional (B12), and toxic (mercury, cobalt, and ethambutol-related) optic neuropathies and inherited disorders affecting mitochondrial function such as Leber hereditary optic neuropathy and dominant optic atrophy. The value of comprehensive electrophysiological phenotyping in syndromic diseases is highlighted in cases of *SSBP1-*related disease and ROSAH (Retinal dystrophy, Optic nerve oedema, Splenomegaly, Anhidrosis and Headache). The review highlights the value of different electrophysiological techniques, for the purposes of differential diagnosis and objective functional phenotyping.

## Abstract (zh)

本文介绍了与神经眼科临床相关的主要视觉电生理诊断试验, 包括视觉诱发电位(VEP), 以及全视野ERG, 图形ERG以及多焦ERG(ffERG；PERG；mfERG)。电生理学检查的基本原则以及对获得性和遗传性视神经病变, 以及与视神经病变需要鉴别的其它视网膜病变, 如视锥细胞与黄斑营养不良、副肿瘤和血管视网膜病变等。VEP和PERG在脱髓鞘、缺血、营养性(B12)和毒性(汞、钴和乙胺丁醇相关)视神经病变以及影响线粒体功能的遗传性疾病的诊断中起到了重要作用, 如Leber遗传性视神经病变和显性视神经萎缩。在SSBP1相关疾病和ROSAH(视网膜营养不良、视神经水肿、脾肿大、多汗和头痛)的病例中, 电生理改变在全身性疾病中的诊断性价值尤为突出。本综述强调了不同的电生理技术在鉴别诊断和客观的视网膜功能以及疾病表型方面的价值。

## Introduction

Symptoms and signs associated with retinal and post-retinal pathology are often non-specific and can pose a diagnostic challenge, with many cases presenting with blurred vision, defective colour vision, visual field defects, a relative afferent pupillary defect (RAPD), and disc swelling/pallor. Precise phenotyping may be confounded by the limitations of subjective psychophysical testing and assessments e.g., visual acuity may be preserved despite significant retinal or optic nerve dysfunction. Electrodiagnostic tests provide objective methods which can localise and characterize dysfunction along the whole of the retino-cortical pathway, resulting from abnormalities evident on retinal imaging or neuroradiology, or “subclinical” lesions that may affect function in the absence of fundus or structural changes. This review outlines the main electrodiagnostic methods relevant to neuro-ophthalmic practice. Examples are shown to illustrate common clinical applications and the principles of interpretation, as well as highlighting some rare acquired and inherited causes of optic neuropathy and syndromic disease.

## Overview of electrodiagnostic procedures

Electrodiagnostic procedures are commonly performed according to the published standards and guidelines of the International Society for Clinical Electrophysiology of Vision (ISCEV; available at www.ISCEV.org), to facilitate meaningful interpretation and inter-laboratory communication. Detailed descriptions of standardised methods [1–5] and common clinical applications including those for retinal disease [6] are beyond the scope of this review, but the main tests are outlined below.

The visual evoked potential (VEP) measures brain activity in the visual cortex elicited by a visual stimulus, allowing an objective evaluation of retino-cortical visual pathway function. There are three types of ISCEV-Standard VEP [1]. The pattern reversal VEP (PVEP) is a response to a reversing checkerboard and is generally the most sensitive in the assessment of optic nerve dysfunction but is of less value if stimulus optics are compromised e.g., by media opacity or an inability to fixate. The pattern appearance-disappearance VEP (PA-VEP), also referred to as onset-offset VEP, is a response to a checkerboard that alternates with a diffuse background (alternation between high and zero contrast), without a change in mean luminance, and has specific applications e.g., in the assessment of patients with nystagmus, or to evaluate suspected functional visual loss. The flash VEP (FVEP) measures the cortical response to a diffuse flash and is used to assess young children or those unable to fixate, in patients with significant media opacity, and can also be a useful complement to the PVEP, especially when dysfunction is severe. All three types of VEP are recorded monocularly, using a midline occipital scalp electrode, and can localise dysfunction anterior to the optic chiasm. An inter-ocular comparison of multichannel VEPs is essential to localise chiasmal and retrochiasmal dysfunction. These recordings require a transverse arrangement of at least 3 and often 5 occipital electrodes, positioned precisely across the occiput and incorporating the midline position [1].

VEP abnormalities are a feature of both post-retinal dysfunction and dysfunction occurring “upstream” in the visual pathway, at the macula or retina, in some cases in the absence of visible or obvious fundus abnormalities. Macular testing, and sometimes retinal testing, is therefore of pivotal importance to enable reliable interpretation of a VEP abnormality.

The electroretinogram (ERG) measures retinal responses to either a contrast change or a focal or global luminance change. Methods standardised by ISCEV include the pattern ERG (PERG) [5], the multifocal ERG (mfERG) [3] and the full-field ERG (ffERG) [2].

The ISCEV Standard PERG is a response to an alternating checkerboard stimulus and has two major components of diagnostic importance i.e., P50 and N95 [5]. The P50 component is closely dependant on macular cone system function but is largely generated by retinal ganglion cell (RGC) activity (approximately 70% contribution) and partly in more anterior retinal structures such as the bipolar cells (approximately 30%) [7]. Despite the major RGC contribution, the PERG P50 component has a well-established clinical value in the assessment of macular cone system function [6, 8]. The N95 component originates wholly in the RGCs and the N95:P50 ratio provides a clinically useful measure of RGC function. The PERG P50 component may be delayed or reduced or both in the presence of macular dysfunction, typically with proportionate reduction in the N95:P50 ratio. In RGC dysfunction, the N95:P50 ratio may be selectively reduced with preservation of P50; in severe cases however, P50 may be reduced by up to 70%, usually accompanied by abnormal shortening of P50 peak time, reflecting loss of the RGC contribution to the P50 peak. The photopic negative response [9] (PhNR - see below) is a late component of the ffERG and may be used to assess global RGC function but is less sensitive than the PERG to dysfunction primarily affecting the papillomacular bundle.

The ISCEV Standard ffERG [2] is a mass response of the neurosensory retina to uniform flashes of light delivered by a wide field (ganzfeld) stimulator. A range of white flashes of different strengths and frequencies are delivered under dark-adapted (DA) and light-adapted (LA) conditions and are used to selectively or preferentially stimulate the rod or the cone systems, with different ERG components used to assess generalised outer and inner retinal function. Under DA conditions, a dim white flash (0.01 cd·s·m^−2^) elicits a positive polarity response (the DA0.01 ERG), which depends on rod photoreceptor function, but arises in the rod bipolar cells, providing a selective suprathreshold measure of rod system function. Stronger flashes (3 and 10 cd·s·m^−2^) are used to evoke the DA3 and DA10 ERGs, each characterised by negative a-waves and positive polarity b-waves. The a-waves largely reflect rod photoreceptor function; the b-waves primarily arise in rod bipolar cells. It is highlighted that DA3 and DA10 ERGs are not selective and there is a small, dark-adapted cone system contribution to both, with a relatively greater contribution from rod photoreceptors to the stronger flash (DA10) ERG a-wave. High frequency oscillatory potentials are superimposed on the ascending limb of the b-wave, generated by amacrine cell signalling within the inner retina. After 10 min light adaptation, the LA ERGs are recorded to a flash strength of 3 cd·s·m^−2^, including the LA 30 Hz flicker ERG and LA3 (single flash “cone”) ERG, in the presence of a photopic background (30 cd·m^−2^). Both types of LA ERG depend on functioning cone photoreceptors, but largely arise in On and Off bipolar cells; the LA3 ERG a-wave reflects mainly cone Off bipolar cell activity with a small cone photoreceptor contribution, and the b-wave reflects summed On and Off bipolar cell contributions.

Full-field ERGs are helpful in determining if there is global retinal involvement, because most cones and rods are located in the retinal periphery and therefore if dysfunction is confined to the macula, both DA and LA ffERGs are typically within normal reference ranges. It is highlighted that ffERGs can be used to assess the nature and severity of generalised rod and cone system function, as well as localising dysfunction to the level of the outer or inner retina (or both). Only a few retinal disorders have pathognomonic ffERG abnormalities [10–12] and diagnostic yield is typically greatest when interpreted in clinical context.

The ISCEV Standard mfERG [3] measures the retinal response to a stimulus array of 61 or 103 modulated hexagonal stimulus elements, covering 40–50 degrees of the central visual field. Each element is illuminated in a fast “pseudo-random” (irregular but pre-determined) m-sequence and a mathematical algorithm then extracts the electrical signals associated with each hexagon. These signals mainly originate in cone On and Off bipolar cells and allow spatial localisation of cone system function across the posterior pole. The mfERG has higher spatial resolution but is more affected by fixation than the PERG and the two tests provide complementary information, the former being a response to local changes in luminance, whereas the PERG is primarily driven by change in contrast. There is no RGC contribution to the ISCEV Standard (1st kernel) mfERG components.

Retinal function can be further studied using non-standard ffERG techniques, currently described and published as extended protocols. These include the ISCEV extended protocol to measure the photopic negative response (PhNR) [9], a component of the ERG that follows the b-wave of the LA single flash ERG, used to assess generalised RGC function, and as such, important in neuro-ophthalmology. The PhNR has proven useful in studies on glaucoma [13–17] and other optic neuropathies [18–22]. The ISCEV extended protocol for the PhNR recommends the use of a red flash on a blue background. However, a study on a large heterogeneous group of patients [23] showed that the PhNR component of the ISCEV Standard LA3 (single white flash) ERG, has only slightly less sensitivity and similar specificity, highlighting the potential convenience of analysing routinely recorded LA3 ERGs for RGC assessment. Other extended protocols include VEP methods to estimate visual acuity, of value in cases of unexplained or functional visual loss [24], extensively reviewed elsewhere [25].

## Retinal masquerades of optic neuropathy

Symptoms such as visual acuity loss, colour vision defect and visual field loss are common in both maculopathies and optic neuropathies. Disc pallor is also common to optic nerve disease and many retinal pathologies, especially in advanced or late-stage disease, and a relative afferent pupillary defect (RAPD) may occur in both if dysfunction is unilateral or asymmetrical.

**Fig. 1:**
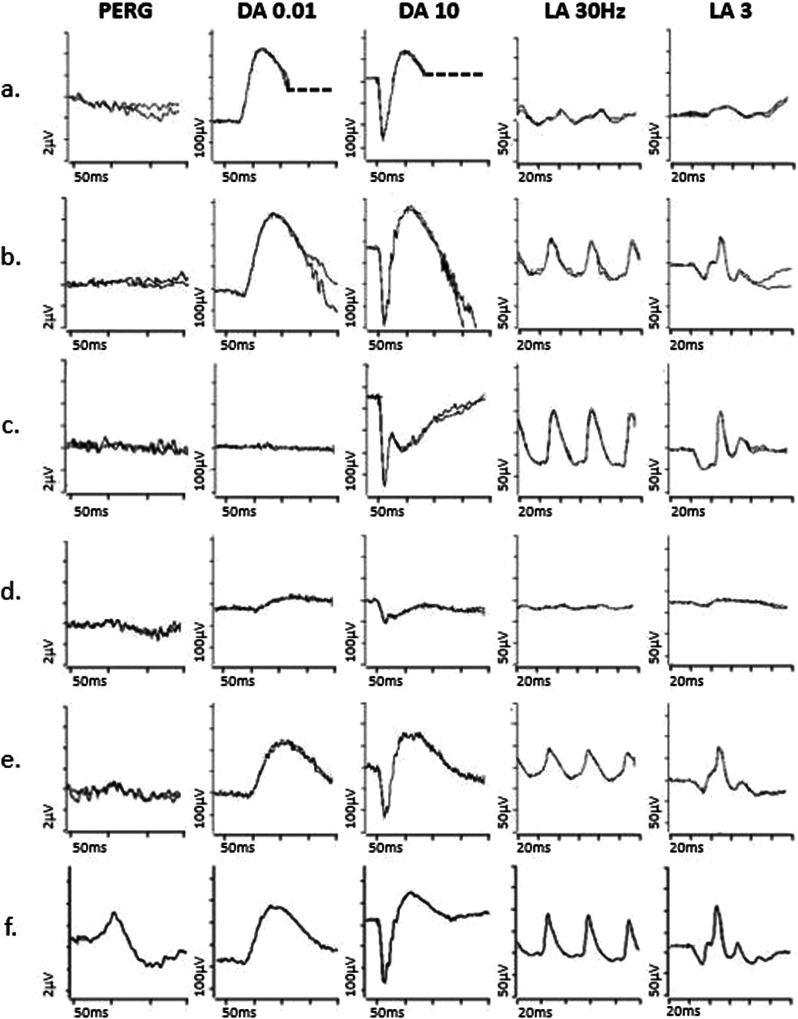
Electrophysiological findings in cases of retinal pathology. Examples of pattern ERGs and full-field ERGs are shown from one eye of a patient with cone dystrophy (**a**), macular dystrophy (**b**), melanoma associated retinopathy (**c**), from right and left eyes of a patient with a right central retinal artery occlusion (**d,** **e**) and from a representative normal subject for comparison (**f**). Recordings showed a high degree of inter-ocular symmetry in all but the patient with a central retinal artery occlusion. All patient traces are superimposed to demonstrate reproducibility. The DA 0.01, DA 10 and LA3 ERGs include a 20 ms pre-stimulus delay. The patient with cone dystrophy has abnormal LA ERGs consistent with generalised (peripheral) cone system dysfunction, with an undetectable PERG P50 component in keeping with severe macular involvement (**a**). In macular dystrophy PERG P50 is undetectable, and ffERGs are normal (**b**), indicating dysfunction confined to the macula. In melanoma associated retinopathy the ffERGs are pathognomonic for generalised On bipolar cell dysfunction, including an electronegative DA10 ERG and specific distortions of the LA 3 ERG waveform (**c**). In CRAO there is unilateral severe generalised inner retinal dysfunction of rod and cone systems, manifest as an electronegative DA10 ERG, with severe LA ERG abnormalities, reflecting severe inner retinal dysfunction (including On and Off bipolar cell dysfunction); of note, patients with long-standing CRAO frequently have additional reduction of the dark adapted (DA3 and DA10) ERG a-wave amplitudes (although relatively mild compared with b-wave reductions). A significant loss of the normal dark-adapted cone system contribution to the DA3 and DA10 ERGs (normally mixed rod and cone system responses) is likely contributory, but the exact mechanism is unknown.

Disc pallor is often a feature of cone and cone-rod dystrophies, in some cases occurring in the absence of other fundus signs. Full-field ERGs can make the distinction, revealing LA ERG+/− DA ERG abnormalities consistent with generalised cone dysfunction (Fig. 1a) or cone more than rod system dysfunction. There is typically early and severe macular dysfunction, sometimes occurring before or without visible signs of maculopathy, which may be assessed using a PERG (Fig. 1a) or mfERG, either of which can be used to inform interpretation of an abnormal VEP.

Pathologies such as resolved central serous chorioretinopathy and occult maculopathies (including *RP1L1*-related occult macular dystrophy) may be characterised by a normal fundus appearance and can be mistaken for an optic neuropathy; in these cases, pattern reversal VEPs are likely to be abnormal, but because of macular dysfunction. If dysfunction is confined to the macula, the ffERGs are normal (Fig. 1b), highlighting the importance of complementary testing with the PERG or the mfERG.

Rapidly progressive loss of vision can occur in paraneoplastic and non-paraneoplastic autoimmune retinopathy (AIR), which often present with normal/near-normal fundi, but with significant retinal dysfunction, evident on ERG testing [26]. The ERG abnormalities show wide variation that likely depends on the antigen, underlying mechanism, and stage of disease, but in carcinoma associated retinopathy (CAR) there is commonly severe rod and cone photoreceptor dysfunction or preferential involvement of cones, and in some cases inner retinal dysfunction [27]. Notably, melanoma associated retinopathy (MAR), rare cases of CAR and even certain non-paraneoplastic AIRs are characterised by ERGs that are pathognomonic for generalised On bipolar cell dysfunction, even in the absence of OCT abnormalities, resulting in an electronegative DA strong flash ERG and other waveform distortions (Fig. 1c); these changes are a phenocopy of ERGs seen in complete congenital stationary night blindness (CSNB) [27–30] and the history will usually help differentiate. Furthermore, rare cases of *CACNA1F*-related incomplete CSNB may also be associated with optic atrophy [31] and this needs to be considered even if presentation is typical for incomplete CSNB. There is phenotypic overlap between non-paraneoplastic AIR and acute zonal occult outer retinopathy (AZOOR)-type disorders [27]. AZOOR can cause a scotoma and retinal dysfunction in one or both eyes, but often with asynchronous and asymmetrical presentation and far more severe than suggested by the fundus abnormality, if present. There is frequently persistent photopsia within the scotoma and there is a high incidence of an enlarged blind spot. The mfERG can help detect or characterise the scotoma. Full-field ERG abnormalities vary and generally include a delay in the LA 30 Hz ERG [32], but there can be a broad spectrum of DA and LA ERG abnormalities [33].

The acute stages of a central retinal artery occlusion (CRAO) or branch retinal artery occlusion (BRAO) typically cause unilateral retinal pallor and oedema that resolve, with subsequent development of disc pallor. However, if the patient fails to notice or report the initial episode, a pale disc can be the presenting feature e.g., in some cases central vision may be preserved due to a cilioretinal artery perfusing the macula, or the effects of the arterial occlusion may go unnoticed in a densely amblyopic eye. Ischaemic optic neuropathy may be suspected (see later). In CRAO there is an “electronegative” DA strong flash ERG (b:a ratio <1) and marked LA ERG abnormalities, in keeping with selective or preferential inner retinal rod and cone system dysfunction (Fig. 1d). The outer retina is perfused by the choroid, and photoreceptor function and consequently the dominant rod-driven contributions to the ERG a-waves are typically relatively preserved.

Posterior scleritis often causes diplopia, vision loss, and eye pain and can therefore be mistaken for optic neuritis, especially if the only fundus abnormality is a swollen optic disc; associated uveitis, if present, may cause retinal dysfunction evident on ffERG testing.

The phenotypic spectrum of some genetically determined syndromes can include both retinal dystrophy and optic atrophy, and several examples are outlined below, highlighting the importance of complementary electrophysiological evaluations of outer retinal and RGC/optic nerve function.

## Primary retinal ganglion cell pathology and inherited optic neuropathies

Many inherited optic neuropathies cause primary RGC pathology; they are an important cause of sight loss in adults and children, affecting approximately 1 in 10,000 people [34] in the UK. The characteristics of RGCs make them particularly vulnerable to mitochondrial dysfunction, due to their long axons which rely heavily on energy from mitochondria [35, 36]. As a result, inherited optic neuropathies cause irreversible loss of RGCs, leading to optic nerve degeneration, disc pallor and vision loss, although severity can vary. Pathogenic variants responsible for these disorders have been identified in both mitochondrial and nuclear DNA [37–39], the two most common being Leber hereditary optic neuropathy (LHON) and autosomal dominant optic atrophy (DOA), and other rarer hereditary mitochondrial eye disorders,  which are emerging with expanded genetic testing.

### Leber hereditary optic neuropathy

LHON is a primary mitochondrial DNA (mtDNA) disorder and about 90% of cases are caused by one of three specific mutations in the mitochondrial respiratory chain [m.3460 G > A (MT-ND1), m.11778 G > A (MT-ND4) and m.14484 T > C (MT-ND6)] [40–43]. LHON typically begins with painless, subacute vision loss in one eye, with fellow eye involvement within 3–6 months. During the initial phase, a dense central or centro-caecal scotoma develops, and vision deteriorates within a few days, typically to worse than 6/60. In a few cases, in patients with the m.14484 G > A mutation, there may be spontaneous partial recovery of visual acuity. The optic disc in the acute stage is usually swollen and hyperaemic, with some degree of tortuosity of the central retinal vessels, but it may appear normal. RGC loss starts in the papillomacular bundle, leading to temporal atrophy of the optic nerve head in the early phase and progressing to diffuse disc pallor in the chronic stage.

**Fig. 2:**
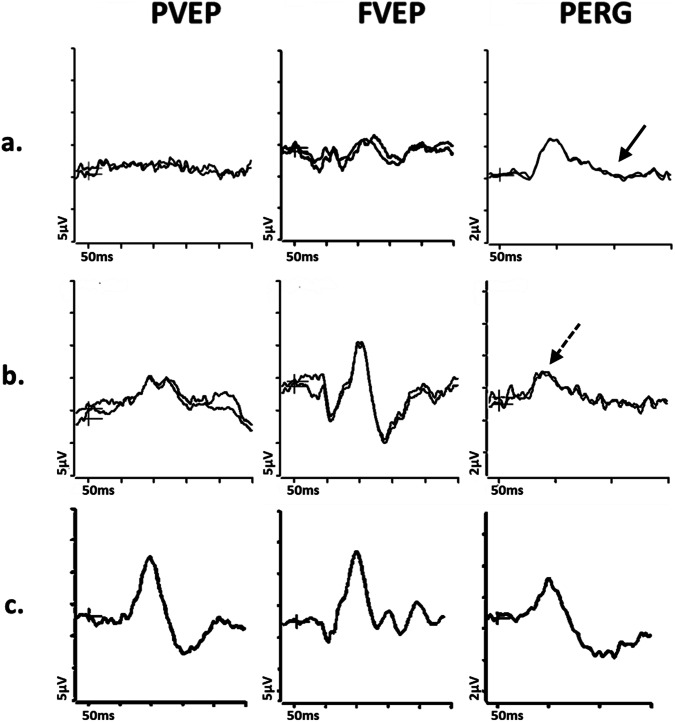
Electrophysiological findings in cases of Leber hereditary optic neuropathy and Dominant optic atrophy. Examples of pattern reversal VEPs (PVEP), flash VEPs (FVEP) and pattern ERGs (PERG) are shown from one eye of a patient with (**a**) Leber Hereditary Optic Neuropathy (LHON); **b** Dominant Optic Atrophy; and **c** from a representative normal subject for comparison. In these cases recordings showed a high degree of inter-ocular symmetry and are shown for one eye only. All patient traces are superimposed to demonstrate reproducibility. The patient with LHON (**a**) has an undetectable PVEP and the FVEP severely reduced with additional waveform distortion, consistent with severe optic nerve dysfunction. The PERG shows P50 preservation of amplitude but mild shortening of peak time and the N95 component is selectively reduced (solid arrow) in keeping with severe macular retinal ganglion cell dysfunction. The patient with DOA (**b**) shows a subnormal and distorted PVEP, preserved FVEP and reduction in the PERG N95:P50 ratio, with additional P50 peak time shortening (arrow with broken lines), consistent with early retinal ganglion cell involvement.

In acute LHON, the PVEP is usually undetectable or severely abnormal, the PERG N95:P50 ratio is reduced (Fig. 2a) and P50 peak time is frequently abnormally shortened [19], both PERG features suggesting primary RGC pathology rather than retrograde axonal degeneration, the latter taking a minimum of 4–5 weeks. Recent evidence suggests additional dysfunction in the macular cone system in LHON cases [44], which may have implications for monitoring disease progression and treatment effectiveness. Furthermore, some LHON mutation carriers or presymptomatic patients show abnormal VEPs and the PhNR may be another useful marker for RGC cell abnormality [18], as it may detect changes before structural abnormalities appear on OCT scans. However, the PERG is a more sensitive measure of RGC dysfunction in the early stages when the papillomacular bundle is primarily affected.

### Dominant optic atrophy

DOA is caused by mutations in the nuclear DNA, mainly involving *OPA1*, which encodes proteins involved in electron transport and ATP synthesis in the mitochondrial inner membrane [45]. There is typically insidious and slowly progressive worsening of vision from childhood and development of bilateral optic disc pallor.

The electrophysiological findings in DOA are usually far milder than in LHON in the early stages (Fig. 2b), but severity varies and the PVEP major positivity may be delayed and of low amplitude, with additional waveform distortion (such as a broad or bifid shape with an anomalous early initial positive peak). The PERG N95:P50 amplitude ratio may be reduced before the PVEP abnormalities occur, consistent with relatively early RGC involvement. In advanced cases, the PERG P50 can show a shortening of peak time and reduction in amplitude. The PhNR can be reduced in some, in keeping with global RGC dysfunction, although sensitivity is limited by the early and more severe involvement of the papillomacular bundle. Some studies have shown abnormal ffERG oscillatory potentials, suggesting mild inner retinal involvement more anterior to the RGCs [20].

### Optic neuropathies associated with systemic features

Mitochondrial disease can cause optic atrophy in isolation or can be associated with systemic involvement. For example, Wolfram syndrome is an autosomal recessive disorder caused by mutations in *WFS1* or *CISD2* [46, 47], and may be characterised by Diabetes Insipidus, Diabetes Mellitus, Optic Atrophy, and Deafness (DIDMOAD), although only about 50% of affected individuals have the full spectrum of clinical features [48, 49]. There is also an autosomal dominant “Wolfram-like” syndrome with pathognomonic OCT findings, showing splitting or lamination of the outer plexiform layer at the macula [50]. The PERG and PVEP findings in autosomal recessive and autosomal dominant Wolfram syndrome are consistent with severe RGC dysfunction including cases with a grossly abnormal PVEP waveform shape [51]. There may be ffERG evidence of additional retinal dysfunction in cases with significant diabetic retinopathy.

Although optic atrophy is common in mitochondrial diseases, there are syndromes caused by mtDNA mutations in which optic neuropathy is a rare manifestation, and other ocular findings are more common, including a pigmentary retinopathy, RPE atrophy, or ocular motility issues; a typical example is chronic progressive external ophthalmoplegia (CPEO), and in these cases, serial ERGs can be informative in guiding the diagnosis and helping exclude other forms of retinopathy, that may have a different clinical course.

### Optic neuropathies associated with retinopathy

**Fig. 3:**
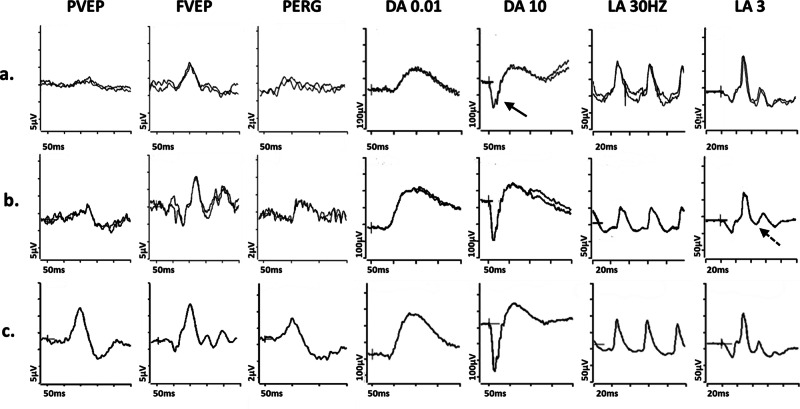
Electrophysiological findings in *SSBP1*- and *ALPK1*- related pathologies. Examples of pattern reversal VEPs (PVEP), flash VEPs (FVEP), pattern ERGs (PERG) and full-field ERGs are shown from one eye of a patient with (**a**) *SSBP1*-related pathology; **b** *ALPK1*-related pathology (ROSAH syndrome); and (**c**) from a representative normal subject for comparison. Recordings showed a high degree of inter-ocular symmetry and are shown for one eye only. All patient traces are superimposed to demonstrate reproducibility. The DA 0.01, DA 10 and LA3 ERGs include a 20 ms pre-stimulus delay. The patient with *SSBP1*-related disease (**a**) shows a subnormal PVEP, a borderline FVEP and reduction in the PERG N95:P50 ratio, with additional P50 peak time shortening, in keeping with macular retinal ganglion cell dysfunction; the DA ERGs are subnormal, with reduction (solid arrow) in the DA10 a-wave, which localises dysfunction at the level of the photoreceptors; LA ERGs are borderline normal. The patient with *ALPK1*-related pathology (**b**) shows a subnormal and delayed PVEP, a normal FVEP and reduction in the PERG N95:P50 ratio, in keeping with macular retinal ganglion cell dysfunction; the DA ERGs are borderline normal; LA ERGs are subnormal, indicative of generalised cone system dysfunction, with additional PhNR attenuation (arrow with broken lines), suggestive of global retinal ganglion cell loss.

In some disorders both optic neuropathy and retinopathy coexist: an *ACO2* mutation has been linked to optic atrophy, vessel narrowing, and retinal degeneration [52], and patients with pathogenic missense variants in *SSBP1* often have optic atrophy, vessel narrowing, and pigmented retinal changes, although there is wide variation [53]. The pigmentary changes may be subtle and the role of comprehensive electrophysiological assessment of retinal function is highlighted; typically, affected individuals show evidence of RGC dysfunction, with additional ffERG abnormalities in some, consistent with a photoreceptor dystrophy (Fig. 3a). *FDXR*-associated disease is a phenotypically heterogeneous disorder with retinal dystrophy being recently reported as a novel ophthalmic feature [54–56]. A preliminary recent report has suggested that biallelic pathogenic *NSUN3*-variants are associated with a wide phenotypic spectrum, ranging from isolated optic neuropathy to syndromic mitochondrial disease, including a case with additional retinopathy [57].

Another example of pathology that involves both optic nerve and retina is ROSAH (Retinal dystrophy, Optic nerve oedema, Splenomegaly, Anhidrosis and Headache) syndrome [58, 59]; this disease is an autosomal dominant disorder caused by mutations in *ALPK1* gene and whilst the acronym highlights some of the features that can be associated with the syndrome, it does not encompass them all.

Although the clinical phenotype is typically characteristic, visible retinal manifestations may be mild and quantifying optic nerve and retinal/macular function (e.g., Fig. 3b) can aid diagnosis and targeted treatment, also informing patient counselling [60].

Combined neurological and retinal pathology are also a feature of juvenile onset Batten disease (juvenile neuronal ceroid lipofuscinosis), a rare inherited neurodegenerative disorder caused by mutations in *CLN3* that primarily affects children and is characterised by the accumulation of lipopigments in various tissues, including the brain. Batten disease typically presents in children with progressive vision loss, cognitive decline, behavioural changes, seizures, and loss of motor skills and as such they are frequently referred to the neuro-ophthalmology clinic. Vision decline may be caused by cortical issues, but in some instances the diagnosis can be suspected based on the ffERG, which may show a low ffERG b:a ratio with relatively mild a-wave loss in the early stages [61, 62]. There is typically early PERG P50 reduction and a high incidence of bull’s eye maculopathy. The ffERG abnormalities vary in severity and responses can be undetectable in severe cases. The importance of early diagnosis is highlighted, given the rapid progression and unfavourable prognosis, to enable appropriate clinical management and support.

## Demyelinating disease

Optic neuritis affects 1–5 per 100,000 people yearly and is a consequence of a demyelinating process, often associated with multiple sclerosis (MS). The main symptoms are vision loss developing over a few days and pain behind the eye that is worse on eye movement or retropulsion. Vision loss can be localised or diffuse and ranges from mild to severe, with 30% of patients experiencing photopsias. The optic disc may be normal or swollen and some patients with MS may also have intermediate or posterior uveitis. It has been reported that a high proportion of patients may be initially misdiagnosed as being affected by optic neuritis, including cases of functional visual loss, ischaemic and other optic neuropathies, and macular and retinal pathologies [63], highlighting the potential value of objective electrophysiological assessment.

**Fig. 4:**
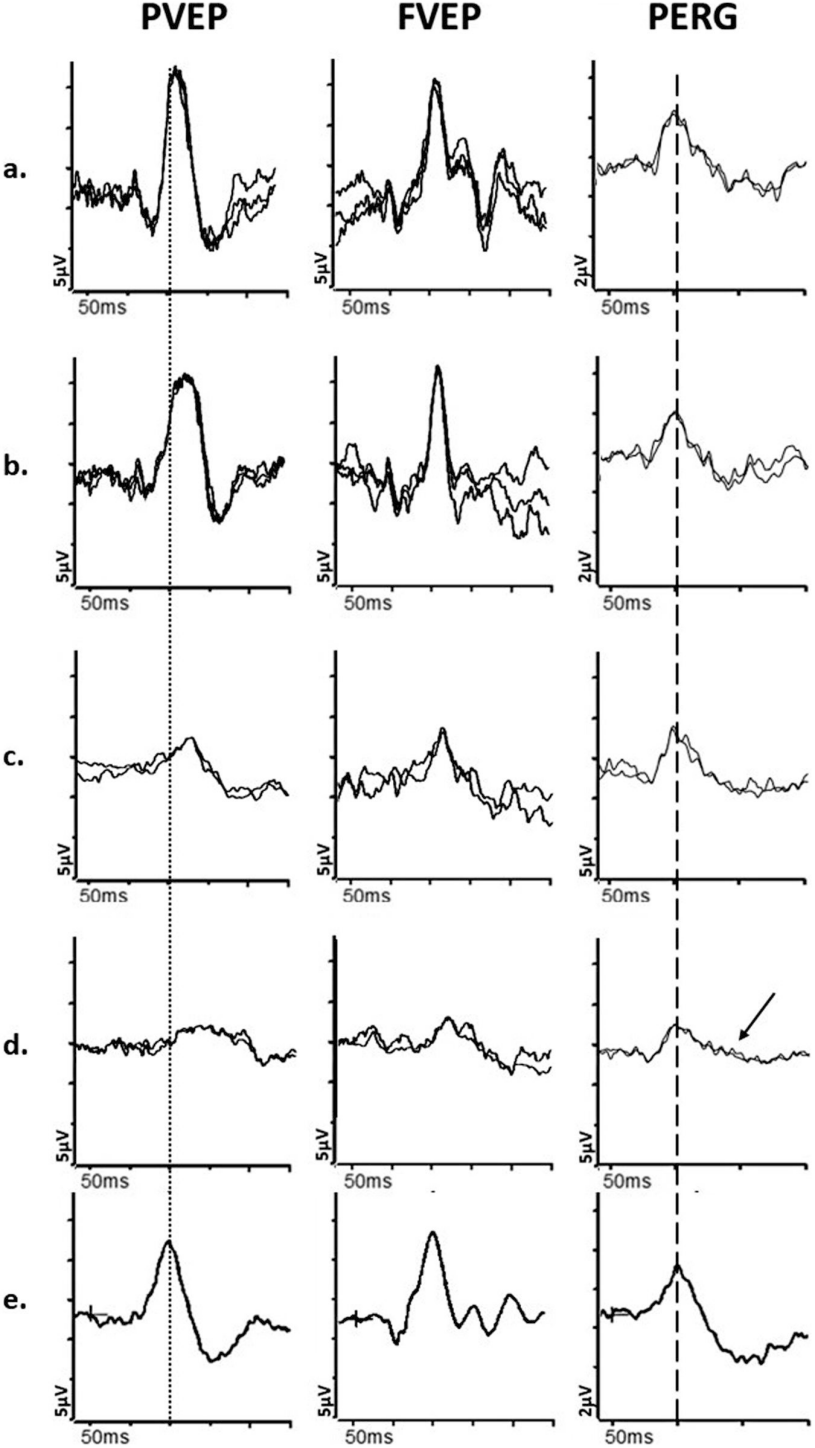
Electrophysiological findings in cases of optic nerve demyelination. Examples of pattern reversal VEPs (PVEP), flash VEPs (FVEP) and pattern ERGs (PERG) from **a** and **b**, a patient with demyelination in the one eye; (**c** and **d**), a patient with demyelination in the left eye and sub-clinical involvement of the right eye; **e** from a representative normal subject for comparison. All patient traces are superimposed to demonstrate reproducibility. Peak time differences are highlighted by the vertical dotted line (PVEP P100) and vertical dashed line (PERG P50). The first patient, with demyelination in the left eye (**b**) has a PVEP P100 of mildly delayed peak time and normal amplitude, consistent with mild optic nerve conduction delay. The flash VEP is normal. The PERG N95:P50 ratio, P50 peak time and amplitude are normal. The asymptomatic right eye (**a**) shows normal PVEP, FVEP and PERG. The second patient’s left eye (**d**) shows a PVEP P100 of markedly delayed peak time and subnormal amplitude, consistent with severe optic nerve conduction delay. Additionally, the FVEP is moderately subnormal and mildly delayed. There is severe PERG N95:P50 ratio reduction (arrow), consistent with severe retinal ganglion cell dysfunction; P50 peak time is normal. The asymptomatic right eye (c) shows a PVEP P100 of moderately delayed peak time and subnormal amplitude, consistent with mild to moderate optic nerve conduction delay and sub-clinical involvement. The right eye PERG is normal.

A typical subacute or chronic feature of optic nerve demyelination is marked delay in the PVEP, often without significant amplitude reduction, persisting after the acute episode has resolved and visual acuity recovered [64]. However, in the acute stages of optic neuritis, the PVEP may be of reduced amplitude and delayed, and the PERG P50 may be subnormal [65, 66], suggesting additional dysfunction anterior to the RGCs, that likely contributes to the acute vision loss [67]. Over a few weeks, as the optic nerve inflammation subsides, the PVEP amplitude tends to recover, but the delay persists (Fig. 4a, b); concomitantly, the pattern ERG P50 improves to normal/near-normal amplitude in the subacute phase. The PERG N95 becomes attenuated in approximately 35% of affected patients [66], thought to be a sign of retrograde degeneration of the RGCs, occurring after a minimum of 4–5 weeks.

In patients with a previous history of optic neuritis, abnormal PVEPs have been reported more frequently than MRI abnormalities [68, 69], and a combined neuroimaging and electrodiagnostic approach may be required to establish the diagnosis. Furthermore, patients with subclinical optic neuritis may show characteristic PVEP delay, and this can be used to support the diagnosis of MS [64, 70] if in combination with non-ocular signs of demyelination. In patients with unilateral optic neuritis, subclinical involvement of the other eye may also explain the lack of a RAPD (Fig. 4c, d).

The ffERG a- and b-waves are typically normal in patients with optic neuritis and MS [71], although there may be PhNR evidence of RGC involvement in chronic cases [22]. Optic neuritis secondary to causes other than MS, like neuromyelitis optica (NMO – anti-aquaporin 4 related disease) and anti-MOG disease have been reported to have ffERG evidence of inner retinal dysfunction in some cases [72], although it remains uncertain whether specific to those disorders. It is also highlighted that there is a relatively high prevalence of uveitis in MS patients compared with the general population. The ffERGs can play an important role in the assessment and management of inflammatory retinal dysfunction and in cases of dual pathology, comprehensive visual electrophysiology (including VEPs) may help determine the cause of visual loss and influence treatment decisions e.g., some biological agents used to treat uveitis may pose a risk of neurological complications [73]. The PVEP has also been used as an objective marker of treatment efficacy e.g., using PVEP peak time improvement as a biomarker for re-myelination [74].

## Ischaemic optic neuropathies

Anterior ischaemic optic neuropathy (AION) is the most common type of acute optic nerve disease in middle-aged and older individuals.

### Non-Arteritic Anterior Ischaemic Optic Neuropathy

Non-arteritic AION accounts for 95% of cases and typically presents with sudden painless loss of vision in one eye, with visual acuity that is usually 6/60 or better, and an altitudinal visual field defect. In the acute stage, there is optic disc swelling, which may be segmental or diffuse, and one or more flame haemorrhages around the optic disc; this is followed by segmental disc pallor in the chronic stage. The aetiology is thought to be reduced blood flow in the short posterior ciliary arteries, leading to damage to the optic disc behind the lamina cribrosa. There are often underlying risk-factors, such as cardiovascular disease, diabetes, sleep apnoea, migraine, certain medications e.g., sildenafil [75] or other phosphodiesterase 5 inhibitors [76], and specific optic disc characteristics (disc-at-risk), such as a small or absent cup, and optic disc drusen. There is also a risk associated with surgical procedures like lumbar spine surgery [77, 78] and intraocular surgery [77–79] (including uncomplicated cases). On occasion, non-arteritic AION can occur in younger patients and can be mistaken for optic neuritis [80, 81].

**Fig. 5:**
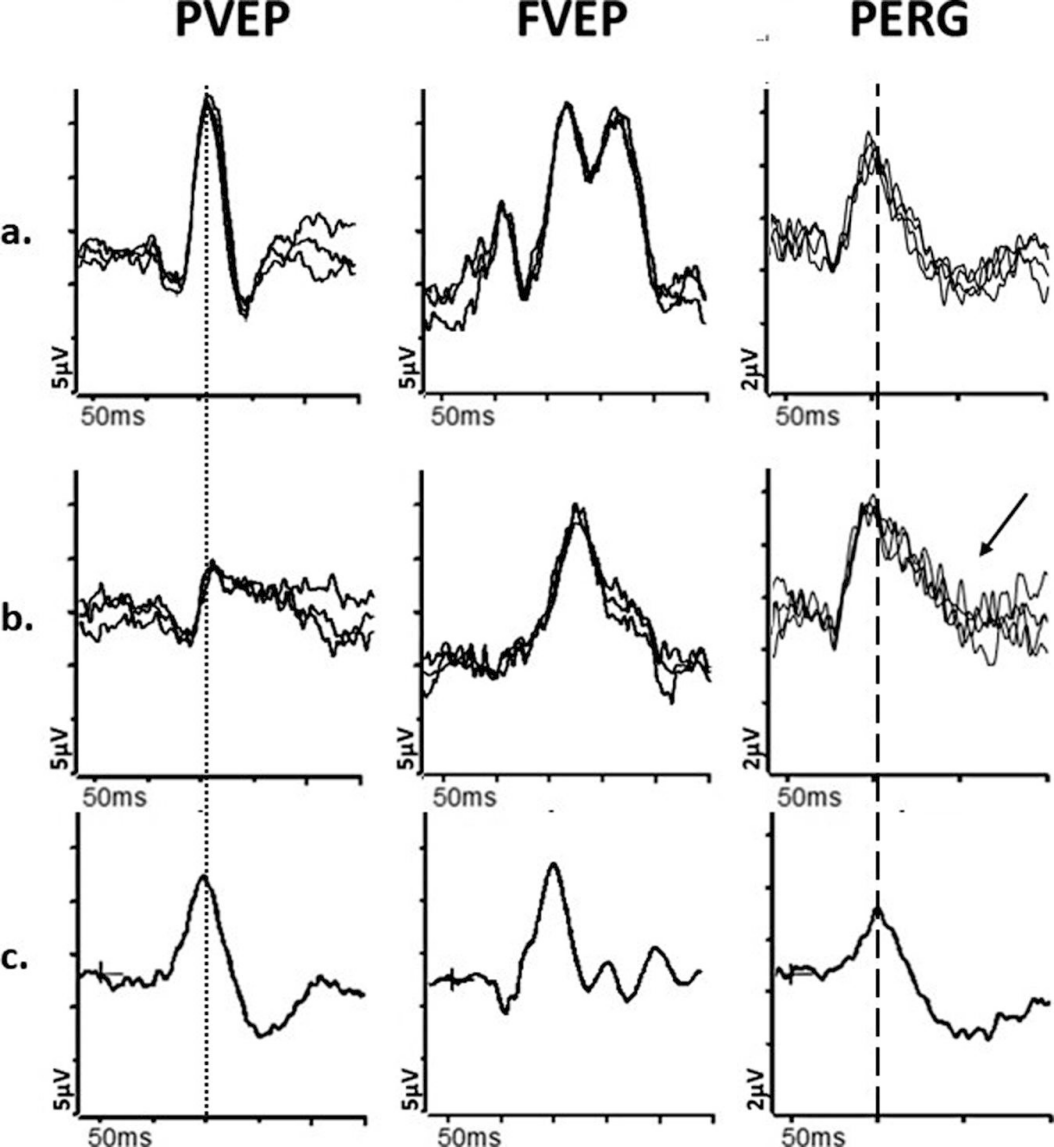
Electrophysiological findings in a patient with non-arteritic ischaemic optic neuropathy. Example of pattern reversal VEP (PVEP), flash VEP (FVEP) and pattern ERG (PERG) from the right (**a**) and left (**b**) eye of a patient with non-arteritic anterior ischaemic optic neuropathy (AION); and from a representative normal subject (**c**) for comparison. All patient traces are superimposed to demonstrate reproducibility. Peak time differences are highlighted by the vertical dotted line (PVEP P100) and vertical dashed line (PERG P50). The left eye with AION (**b**) has a PVEP P100 component of reduced amplitude, poorly formed N135 component, and normal peak time and the FVEP has an altered waveform compared with the contralateral healthy eye, consistent with moderately severe optic nerve dysfunction. The PERG shows P50 preservation but the N95:P50 ratio is markedly reduced (arrow) consistent with marked RGC involvement. The right eye (**a**) is unaffected, with a normal PVEP, FVEP and PERG.

Pattern VEPs in non-arteritic AION typically show reduced amplitude in one eye without significant delay (Fig. 5), which frequently distinguishes it from optic neuropathy secondary to demyelinating disease e.g., it has been reported that interocular peak time asymmetry was, on average, 21 ms for optic neuritis and 3 ms for non-arteritic AION, although with some overlap [82]. Flash VEPs may also show reduced amplitude and delay, but they are generally more variable and less reliable than pattern VEPs. The VEPs in the unaffected eye are typically within the normal range, unlike cases of sub-clinical demyelination; non-arteritic AION in one eye however increases the risk of developing it in the contralateral eye and steps should be taken to reduce any of the mentioned modifiable risk factors the patient may have.

### Arteritic anterior ischaemic optic neuropathy

Arteritic AION causes severe vision loss and is associated with giant cell arteritis (GCA). It is rare before the age of 60 and causes severe vision loss, often accompanied by headaches, jaw claudication (progressively worse pain in jaw muscles secondary to chewing), tenderness over the temporal artery or scalp, weight loss, fatigue, and night sweats. In some cases, there may be intermittent episodes of transient vision loss before the severe loss occurs. In the acute stage, the optic disc is swollen and chalky white, followed by optic atrophy and retinal arteriolar attenuation. The affected and/or fellow eye may show cotton wool spots and may be hypotensive. Of note, patients with GCA are also at risk of a cilio-retinal artery occlusion (central macular dysfunction evident on PERG or mfERG testing with normal ffERG) or central retinal artery occlusion (causing severe inner retinal dysfunction manifest as an electronegative ffERG; Fig. 1d) and AION and retinal arterial occlusions may occur in the same patient [83]; electrophysiology can help establish the extent of additional retinal involvement in suspicious cases. In arteritic AION, there is usually a severely attenuated or abolished PVEP and FVEP. The fellow eye is frequently involved within days or weeks unless aggressive treatment is initiated promptly. Arteritic and non-arteritic AION often ultimately result in PERG N95 reduction [84, 85] (Fig. 5b) and frequently the P50 is also attenuated [66, 86], more often than in optic neuritis, reflecting the extent of RGC involvement, as also demonstrated by the typical loss of RGCs on OCT. There can also be attenuation of the PhNR.

## Nutritional and toxic optic neuropathies

Nutritional deficiency in B complex vitamins [thiamine (B1), riboflavin (B2), niacin (B3), pyridoxine (B6), cobalamin (B12)], folic acid, and proteins with sulphur-containing amino acids may lead to optic nerve pathology. Toxins that cause optic neuropathies include certain medications, organic solvents, metals, cobalt, mercury, and carbon monoxide. Methanol and quinine toxicity may also cause optic atrophy and inner retinal dysfunction. Patients with nutritional and toxic optic neuropathies typically present with similar ophthalmic symptoms and signs, including bilateral vision loss, central or centro-caecal scotomas, colour vision deficits, loss of the papillomacular bundle and optic atrophy. Pathologies may also be multi-factorial e.g., heavy alcohol and tobacco consumption may trigger conversion in LHON mutation carriers [87] or may be associated with a poor diet, malnutrition, and vitamin B deficiency [88]. It is also highlighted that some toxins cause optic nerve dysfunction and/or retinal dysfunction, evident on ffERG testing. Early diagnosis is important in both nutritional and toxic disease, as there may be potential to arrest progression or even restore vision.

**Fig. 6:**
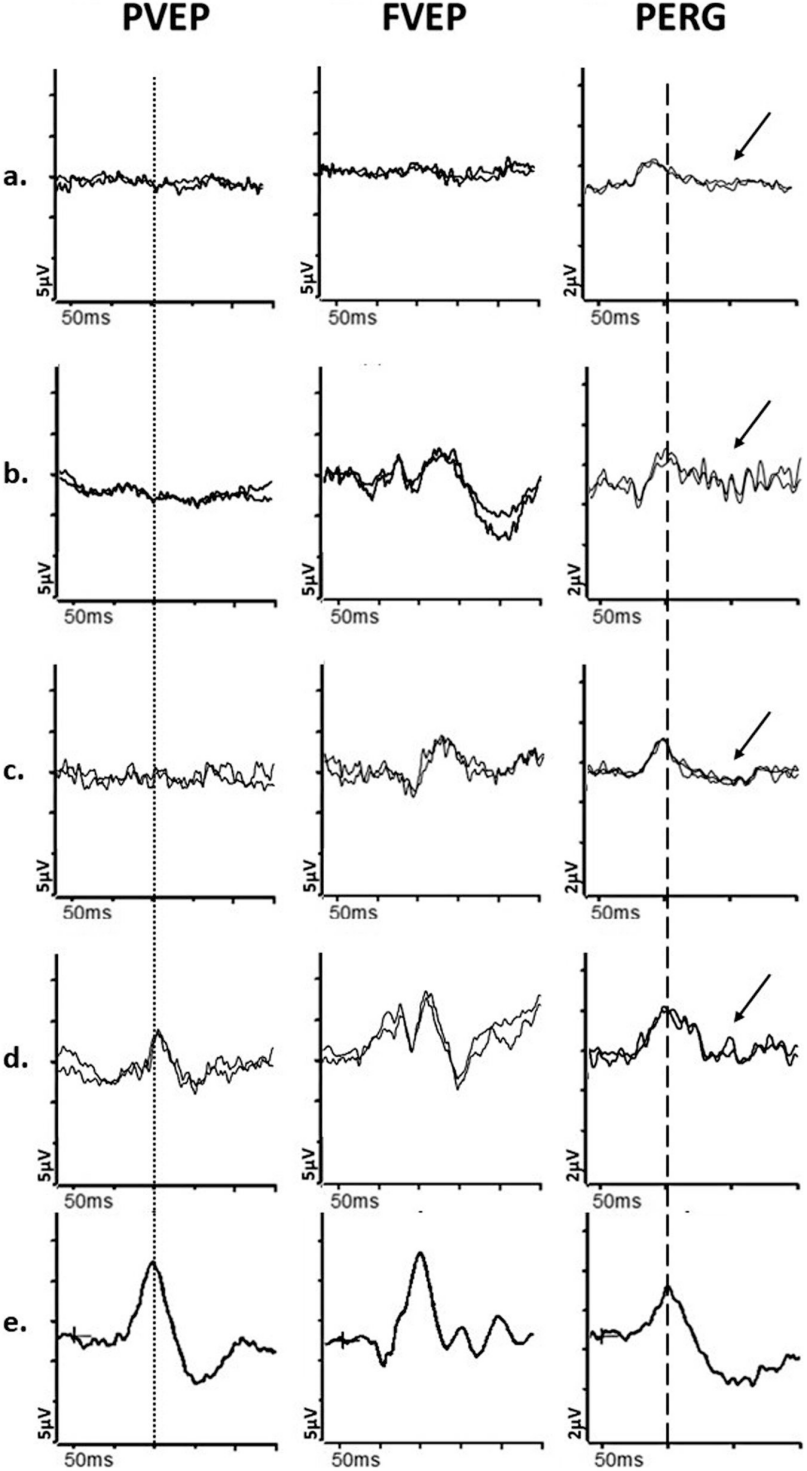
Electrophysiological findings in cases of nutritional and toxic neuropathies. Examples of pattern reversal VEPs (PVEP), flash VEPs (FVEP) and pattern ERGs (PERG) from a patient with chronic alcohol withdrawal syndrome and Vitamin B12 deficiency (**a**); from a patient initially suspected of LHON that had developed cobalt toxicity due to cobalt leaching out from the femoral heads following multiple total hip replacements (**b**); from a patient initially suspected of LHON and subsequently diagnosed with mercury toxicity, demonstrated by high levels of mercury in urine, resulting from a prolonged and restricted diet of fish high in methyl mercury (**c**); from a case of ethambutol toxicity following treatment for lymph node TB (**d**); from a representative normal subject for comparison (**e**). All recordings showed a high degree of inter-ocular symmetry and are shown for one eye only. All patient traces are superimposed to demonstrate reproducibility. Peak time differences are highlighted by the vertical dotted line (PVEP P100) and vertical dashed line (PERG P50). The patient with Vit B12 deficiency (a) has an undetectable PVEP and the FVEP is severely reduced, consistent with severe optic nerve dysfunction. The PERG N95:P50 ratio is severely reduced (arrow); P50 peak time is markedly shortened with marginal reduction in amplitude, consistent with severe RGC involvement. The patient with Cobalt toxicity (**b**) shows an undetectable PVEP and FVEP of reduced amplitude, consistent with moderately severe optic neuropathy; in addition, there is severe reduction of the PERG N95:P50 ratio (arrow), with mild shortening of P50 peak time, consistent with retinal ganglion cell dysfunction. The PERG P50 amplitude is preserved. The patient with Mercury toxicity (**c**) shows moderate PERG N95:P50 ratio reduction (arrow) and shortened P50 peak time, consistent with moderate retinal ganglion cell dysfunction; PERG P50 amplitude is preserved. In addition, the PVEP is undetectable and the FVEP amplitude is of marginally reduced amplitude, consistent with optic nerve involvement. The patient with Ethambutol toxicity (**d**) shows a marginally delayed and subnormal PVEP P100, and normal FVEP, consistent with mild optic nerve dysfunction. The PERG P50 peak time and amplitude are normal but the N95:P50 ratio is reduced (arrow), consistent with retinal ganglion cell involvement.

Visual electrophysiology (Fig. 6) can localise and monitor dysfunction and possible recovery, although VEP and PERG abnormalities are usually non-specific and reflect the severity of optic nerve/RGC involvement. As an example, in typical cases of ethambutol toxicity the PVEP can show symmetrical delay and amplitudes are frequently subnormal, with PERG evidence of RGC involvement in severe cases (Fig. 6d). Disc pallor is usually associated with poor visual prognosis [89] but if toxicity is diagnosed early and cessation of treatment is prompt, visual impairment may be reversed and severe pattern VEP abnormalities may resolve [90].

Cobalt-related optic neuropathy and/or retinopathy, can occur when cobalt-chromium ions are released from a metal hip prosthesis [91]: cobalt-chromium has the potential to interfere with mitochondrial function and as RGCs are particularly susceptible to mitochondrial damage, cobalt can cause RGC dysfunction, which can manifest as PERG and VEP abnormalities (Fig. 6b). Cobalt also stops communication between photoreceptors and second order neurons by blocking calcium-dependent synaptic transmission, potentially damaging both the outer and inner retina, and causing dysfunction evident on ffERG testing. Mercury-related optic neuropathy may occur when excessive amounts are accidentally ingested as part of a fish-based diet (Fig. 6c).

Identifying these and similar causes of optic neuropathies before excessive permanent optic nerve damage has occurred is essential in adequate patient management and electrophysiology can play an important role in ensuring a timely diagnosis is made and in objectively monitoring progression, potential recovery or treatment efficacy [92].

## Chiasmal abnormalities/dysfunction

Multichannel VEPs, that measure the response across all the visual cortex when each eye is stimulated monocularly, are necessary to assess chiasmal and retro-chiasmal dysfunction. Multichannel VEPs reflect the known projection of RGC axons to the occipital cortex, with fibres from the temporal retina going primarily to the ipsilateral cortex, and those from the nasal retina to the contralateral cortex.

### Contralateral predominance/crossed asymmetry

**Fig. 7:**
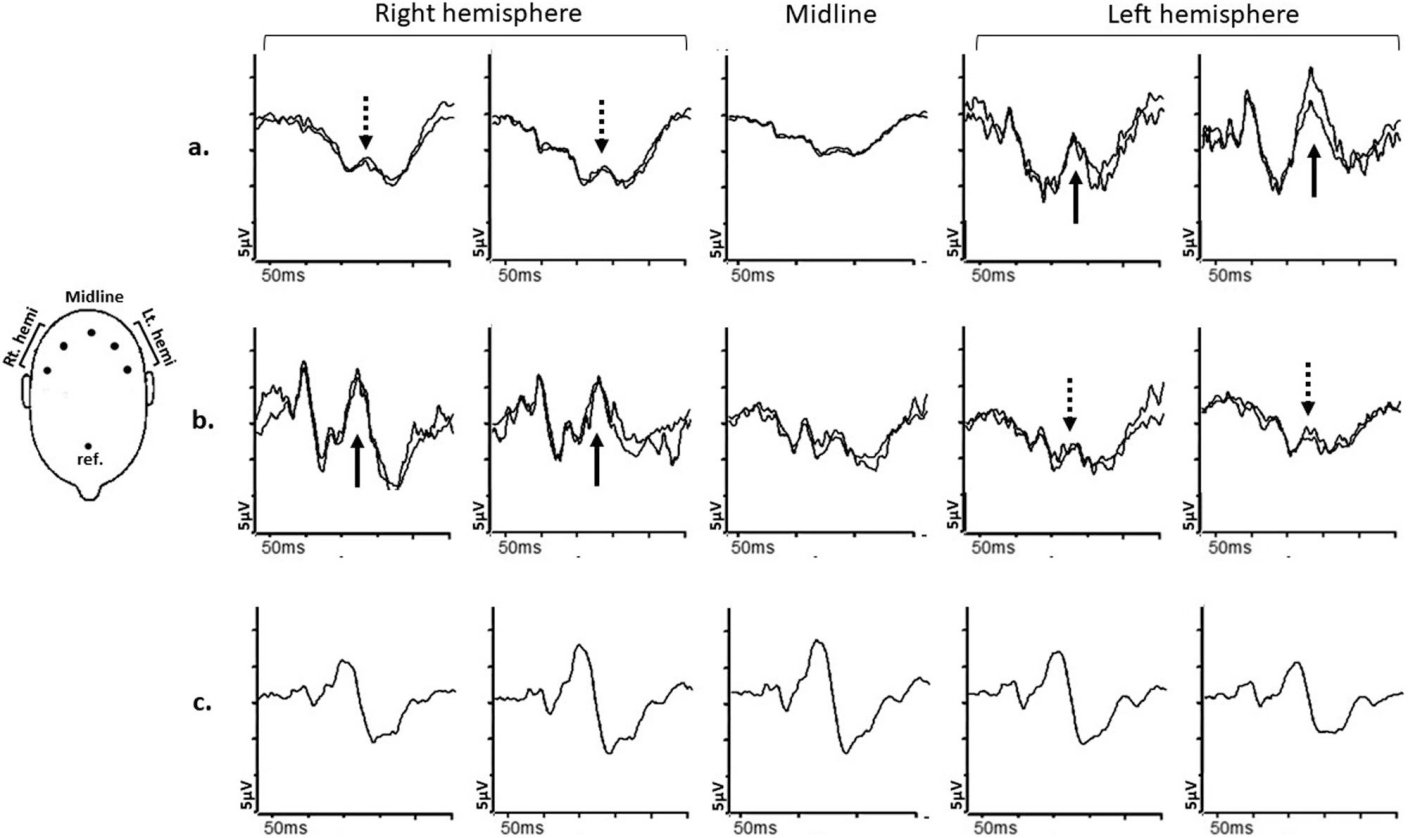
Multichannel pattern onset-offset VEPs in a case of albinism. Recordings **a** and **b**: from a patient with ocular albinism and (**c**): from a representative normal subject for comparison, using five scalp electrodes positioned over the occiput; 2 electrodes over the right hemisphere; two electrodes over the left hemisphere; 1 electrode over the midline. **a** Pattern onset-offset VEPs from stimulation of the right eye of the patient with albinism are present and of significantly larger amplitude from the left hemisphere (bold arrows) compared with the right hemisphere (dashed arrows), consistent with contralateral predominance; VEP responses from stimulation of the left eye (**b**) are present and of significantly larger amplitude from the right hemisphere (bold arrows) compared with the left hemisphere (dashed arrows), consistent with contralateral predominance. **c** VEP responses from the normal subject are of similar amplitude and peak time from the left and right hemisphere.

In albinism or other conditions like *SLC38A8*-related disease, which are inherited and usually stable conditions, most of the nerve fibres in the optic nerve from one eye project to the contralateral hemisphere of the brain i.e., the left hemisphere receives input primarily from the right eye, and the right hemisphere primarily from the left eye [93, 94]. Monocular multichannel pattern appearance (in adults) or flash VEPs (in younger children) can be used to identify this contralateral predominance or “crossed asymmetry” (Fig. 7) and help in the diagnostic process [95], especially when clinical presentation is not characteristic or equivocal [96].

### Compressive lesions

Pre-chiasmal, chiasmal and retro-chiasmal compressive lesions typically cause a slow and gradual loss of vision and/or visual field defects that reflect the location of the lesion. Onset can be more rapid if there is an inflammatory, infective, or aneurysmal element to the space occupying lesion.

If the compression is pre-chiasmal, VEP and on occasion PERG abnormalities are present only in the eye ipsilateral to the compression. The most common optic nerve tumour in children is a glioma, which is frequently associated with neurofibromatosis type 1 [97]. In adults, the most common tumours that affect the visual pathway are pituitary adenomas and optic nerve sheath meningiomas. It is highlighted that flash VEPs are usually less sensitive than PVEPs to optic nerve dysfunction, but rare cases of optic nerve sheath/cavernous sinus meningioma have demonstrated the opposite, with significant involvement of the PVEP only in the latter stages of the disease process [98].

In chiasmal and retro-chiasmal pathology, multichannel VEPs can help locate the site of the lesion, with chiasmal pathology causing ipsilateral predominance to monocular testing; retro-chiasmal pathology will cause VEP abnormalities over the same hemisphere from both eyes, irrespective of stimulation being stimulation is monocular or binocular. The VEP abnormalities are frequently associated with specific visual field defects, including bitemporal field defects and homonymous congruous or incongruous hemianopia or quadrantanopia, dependent on the site of the lesion. It is noted that VEPs can play an important role in monitoring patients with compression of the visual pathways, especially when management is observation (e.g., in slow-growing meningiomas), as dysfunction may be worsened in the absence of neuroimaging changes [99] and may be reversible with prompt intervention. Furthermore, the PERG N95:P50 has been shown on occasion to be a good biomarker of post-operative visual outcome [100, 101].

## Functional visual loss

Electrophysiology is of high value in managing individuals with suspected functional visual loss. Normal findings can help exclude other causes of disease, and relatively mild dysfunction in the context of severe visual loss/symptoms may indicate a functional overlay, in which severity of symptoms is exaggerated. Modified protocols, like simultaneously recording PERGs and PVEPs, can be helpful, as a preserved PERG P50 excludes significant fixation error and defocus, informing PVEP interpretation. It is also useful to actively monitor background EEG intrusion in the VEP, as this may indicate patient drowsiness that frequently shows “paradoxical alpha rhythm”, a typical feature of drowsiness/loss of attention when the eyes are open.

A normal PVEP is difficult to reconcile with a visual acuity of 6/36 or worse, and a detectable flash VEP is inconsistent with no perception of light, but Standard VEP techniques cannot measure visual acuity. An estimation of visual acuity requires specific VEP methods that measure a threshold as an indirect measurement of visual system resolution and have been reviewed extensively recently [24, 25].

It is stressed that normal visual electrophysiology does not rule out all possible underlying pathologies; for example, the PVEP, PERG and mfERG can be preserved with a highly localised foveal lesion, and VEPs can be normal in some cases of cortical blindness related to pathology beyond the visual cortex, and clinical context is of paramount importance.

## Other uses of electrophysiology in neuro-ophthalmology

Lesions of both occipital lobes or higher visual centres can lead to cortical blindness. This can be caused by stroke, tumour invasion, or infections like meningitis. Cortical blindness typically presents with total or partial loss of visual fields, bilateral severe vision loss, and normal eye structure and pupillary responses. The PVEPs are often undetectable or significantly abnormal in occipital lobe lesions but may be normal if the higher cortical centres are the cause of vision loss; ffERGs and the PERG (providing fixation is adequate) are typically normal.

### Congenital optic nerve disease

Congenital disorders of the optic nerve can be unilateral or bilateral, and may be associated with abnormalities in other ocular structures, like the retina, the choroid and/or the iris. The clinical manifestations of congenital optic nerve pathologies, like optic disc coloboma or optic disc hypoplasia, vary, some individuals having relatively minor visual disturbances, whilst others may experience significant vision impairment or even blindness in the affected eye. The level of dysfunction may not always be predictable with clinical examination alone and electrophysiology can help quantify dysfunction of the optic nerve and help establish if other surrounding structures, like the macula or retina, have been involved in the process. Regular evaluations may also help monitor the individual’s visual development and address any emerging issues.

### Amblyopia

In the context of amblyopia, clinical presentation is usually sufficient to establish the diagnosis, but electrophysiological studies can help in uncertain cases, by excluding abnormalities of the retina or optic nerve as a cause of the reduction in vision in the presence of normal/near normal findings, or by evaluating if there is a functional overlay, thereby reassuring the patient and their family and assisting in management.

## Conclusions

Optic neuropathies, maculopathies and retinopathies can present with similar symptoms and signs; electrophysiological tests are rarely specific, but they can help localise the dysfunction and give important diagnostic information about visual function that cannot be otherwise inferred. Performing all tests to International Standard (ISCEV) requirements enables consistent interpretation, meaningful inter-laboratory comparisons, and improved reliability of serial testing. Standardised testing is often fundamental to diagnosis and has played a central role in the functional phenotyping of genetic and acquired disorders. The importance of electrodiagnostic tests in neuro-ophthalmology is likely to increase as novel therapies emerge, for both inherited and acquired optic neuropathies.
